# The Failure Mechanism of the 316 SS Heat Exchanger Tube in the Geothermal Water Environment

**DOI:** 10.3390/ma15228103

**Published:** 2022-11-16

**Authors:** Jike Yang, Chan Li, Yue Pan, Hui Huang

**Affiliations:** 1 Corrosion and Protection Center, University of Science and Technology Beijing, Beijing 100083, China; 2 Nuclear and Radiation Safety Center, Ministry of Ecology and Environment, Beijing 100082, China; 3 China Special Equipment Inspection and Research Institute, Beijing 100029, China

**Keywords:** 316 stainless steel, stress corrosion cracking, heat exchanger tube, failure investigation

## Abstract

In this work, the intrinsic reason for the premature failure of a 316 stainless steel heat exchanger tube in geothermal water environment is disclosed. The chemical composition of the tube was tested, and the microstructure was examined for material inspection. Fracture morphology and secondary cracks were analyzed, and electron backscattered diffraction was applied to explore the crack propagation mode. The corrosion morphology was observed. The electrochemical behavior was studied with cyclic polarization and double-loop electrochemical potentiokinetic reactivation. It is found that the main failure cause was stress corrosion cracking (SCC). Attacked by chloride ions, the tube is susceptible to SCC under the residual stress as a result of the substandard Mo and Ni content. The SCC mechanism is localized anodic dissolution, and the propagation mode is a mixture of transgranular SCC and intergranular SCC.

## 1. Introduction

With the development of the petroleum industry, atomic energy industry, food industry and pharmaceutical industry, the 300 series austenitic stainless steels have been extensively used because of their superiorities in high temperature resistance, formability, mechanical properties and corrosion resistance [1,2,3]. Among them, 316 austenitic stainless steel (SS) has been widely adopted in piping systems, heat exchangers, liquid vessels and other industrial facilities [4,5,6]. However, numerous studies and reports show that the 316 SS has suffered from stress corrosion cracking (SCC) accidents during service in industrial environments in recent years, which causes catastrophic consequences [7].

SCC is a type of environmentally assisted cracking (EAC) under the synergetic effect of three factors including corrosive environment, stress and SCC-susceptible materials [8,9,10,11]. In recent years, the SCC of 316 SS parts has occurred frequently, which is one of the most threatening hazards for industrial production. The cause of SCC failures is owed to the above three factors. As for corrosive environments, it has been widely recognized that austenitic stainless steels are susceptible to SCC in an acidic Cl^−^ environment. Jawwad et al. [12] conducted a comprehensive failure analysis on a failed 316 SS nozzle and sealing disk. They found the failure mode was high-stress regime transgranular SCC promoted by a corrosive environment enriched in Cl^−^ and H_2_S. Martins et al. [13] concluded that the failure cause of the AISI 316 SS downhole pressure memory instrument cover was chloride-induced SCC. Cl^−^ can promote SCC initiation and propagation in industrial environments [3,6]. Cl^−^ can penetrate into passive film and promote the passive film breakdown by occupying oxygen vacancies and dissolving Cr oxides [14,15]. Therefore, pitting corrosion is prone to initiate on the weak area of passive film and forms SCC initiation sites [14,16,17,18]. Cl^−^ can also enrich in the SCC crack tip, destabilize the passive film and promote SCC propagation [19]. 

Residual stress has been reported to cause SCC during the service of 316 SS parts [20,21]. Previous studies have shown that surface processing can cause residual stress, which destructs the passive film of austenitic stainless steels [22,23,24]. The passive film destruction attributed to residual stress enables environmental attack at the surface and induces high SCC susceptibility [25]. Moreover, surface processing can induce internal tensile stress, which results in SCC initiation [26]. Consequently, 316 SS suffers from premature failure under the combined effect of external stress and corrosive environment.

As for susceptible materials, microstructure degradation can lead to SCC failure. Solomon et al. [27] revealed that strain-induced martensitic transformation has a significant effect on the SCC resistance of AISI 316 austenitic stainless steel plate heat exchangers. As proved by Liu et al. [28], the sensitization of 304 SS leads to the occurrence of branching SCC cracks.

There are plenty of research methods for SCC, including simulation methods (e.g., finite element method [29] and phase-field theory [30,31,32]), analytical methods and experimental methods. Simulation methods and analytical methods are useful for mechanism studies based on thermodynamics, kinetics and fracture mechanics. However, with regard to a specific failure case, experimental methods can be more applicable for understanding the characteristics of failure and disclosing the main cause.

Though SCC of 316 SS has been extensively investigated, when encountering specific failure cases, it should be treated differently depending on different situations. Additionally, the detailed determining factors and mechanism need to be clarified for mitigating SCC. In the work, we aimed at an SCC failure case of a 316 SS heat exchanger tube served in the water environment, which ruptured suddenly after one year of service and caused a leakage of geothermal water. Failure analysis was conducted to find out the main cause of failure through a series of experimental analysis. On this basis, the failure mechanism and further suggestions were proposed to provide theoretical support for the failure studies of equipment.

## 2. Experimental Procedures

First, a field investigation of the service condition was conducted, and the failed tube was inspected macroscopically. On this basis, the failure locations were determined and cut off for detailed analysis. The microstructure and chemical composition of the specimens were tested for material inspection. Fracture morphology and cracks were observed for studying the fracture features and mechanism. Electrochemical measurements were performed, and corrosion morphology was carefully observed in order to study corrosion behavior. Based on the results, the failure mechanism was proposed, and the targeted suggestions for preventing failure cases of 316 SS heat exchange tubes were proposed. It should be noted that the research methods in this work also apply to any failure cases related to EAC.

### 2.1. The Field Investigation

**Figure 1:**
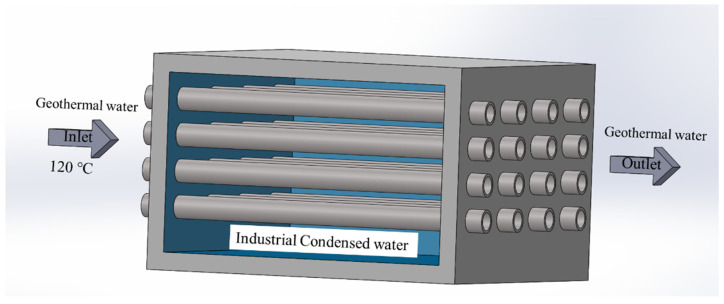
Schematical diagram of the service condition of the convection tube.

The research subject is a failed 316 SS heat exchange tube used to transmit heat from high-temperature geothermal water, and the service condition is shown in Figure 1. Geothermal water about 120 °C flows into the convection tube from the inlet, and the media of the external tube is industrial condensed water. The thermal gradient between the geothermal water and industrial condensed water causes the momentum transfer process. The internal diameter and external diameter are 20 mm and 26 mm, respectively. The tube was in service for about a year before it ruptured, causing a leakage of geothermal water.

### 2.2. Material Inspection

The chemical composition of the failed tube was tested with an ARL-4460 spectrometer, and the microstructure was observed with an OLS5100 confocal laser scanning microscope (CLSM). Specimens for microstructure observation were ground sequentially to 5000# emery paper, mechanically polished with diamond paste with 1.0 μm particle size and chemically etched with solution prepared with 100 mL deionized water + 50 mL hydrochloric acid + 5 g FeCl_3_.

### 2.3. Electrochemical Tests

The traditional three-electrode system was adopted for the electrochemical test, in which the test specimen with an exposing area of 10 mm × 10 mm worked as the working electrode, the platinum sheet with an area of 20 mm × 20 mm as the auxiliary electrode and the saturated calomel electrode (SCE) linked with a salt bridge worked as the reference electrode. The working specimen was sequentially ground to 2000# with emery paper, washed with deionized water and acetone, followed by blow-dry prior to test. Before electrochemical measurements, the potentiostatic polarization of −1.0 V (vs. SCE) was imposed for 200 s to remove oxide film formed in air, and then the open circuit potential (OCP) value with a function of time was recorded until reaching a stable value. All the tests were performed at least three times at ambient temperature.

The double loop electrochemical potentiokinetic reactivation (DL-EPR) method was conducted to characterize the degree of sensitization (*DOS*) of the tube material according to the Chinese National Standard GB/T 29088-2012. Solution treated specimens were used for comparison, and heat treatment was conducted with the specimens cut off the tube by being heated at 1050 °C for 1 h and cooled in water. The test solution was 0.5 mol/L H_2_SO_4_ and 0.01 mol/L KSCN. The specimen was polarized from OCP to 300 mV_SCE_ at a sweeping speed of 6 V/h, and then the potential was scanned back to OCP at the same speed. From the DL-EPR curve, the ratio between the peak current density during the forward scan (*I_a_*) and the peak current density during the reverse scan (*I_r_*) was calculated to quantify *DOS*:

(1) $$DOS=\frac{I_{r}}{I_{a}}\times 100\%$$

Cyclic potentiodynamic tests were conducted for assessing the corrosion resistance. The test solution was 0.1 mol/L NaCl with various pH values of 3, 5, 7, 9, 11 and 13. The cyclic polarization was scanned from −0.1 V versus open circuit potential until the current density reached 0.5 mA/m^2^ or potential exceeded 0.6 V (vs. SCE), then scanned reversely and stopped manually when the curve intersected with the forward scan curve. The pitting potential was defined as the current density at 0.1 mA/cm^2^ and the protection potential was the intersection potential [33].

### 2.4. Observation of Corrosion and Fracture Morphology

Corrosion morphology, fracture morphology and secondary cracks were observed with a JSM-6610LV tungsten filament scanning electron microscope (SEM) after corrosion product removal with ultrasonic cleaning in 10% oxalic acid and the three-dimensional corrosion morphology and corrosion depth were measured by the OLS5100 CLSM. The deepest corrosion depth and the average corrosion depth were analyzed by the Multiple File Analysis Software. The chemical composition of corrosion products was analyzed with Inca X-ac electron energy dispersion spectrum (EDS) equipped on SEM. Some deposits on the tube were analyzed with X-ray diffraction (XRD) with a Smartlab X-ray diffractometer. The scanning rate of XRD was 3°/min, and the scanning range was 10–90°. 

Electron backscattered diffraction (EBSD) was conducted to study the crack propagation mode. Before testing, the specimen for EBSD observation was successively ground to 5000# with emery paper, mechanically polished with diamond paste with the particle size of 0.5 μm, and finally polished to 50 nm with Al_2_O_3_ oxide polishing suspension. The EBSD test was conducted on a Tescan Mira 3 LMH SEM and the signals were collected by an Oxford Instrument symmetry probe. The step size of the overall map is 0.5 μm while the step size of the crack tips is 0.15 μm. After the test, Channel 5 HKL software was used to analyze the raw data.

## 3. Results and Discussion

### 3.1. Preliminary Visual Examination

**Figure 2:**
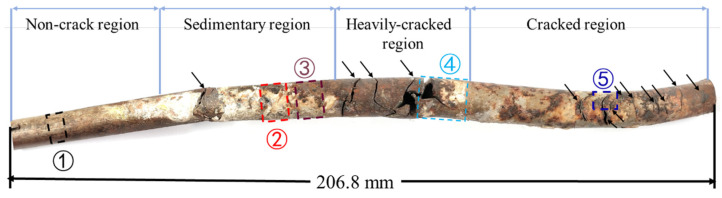
The optical photograph of the failed tube and sampling positions for tests. The black arrows point to the macroscopic cracks on the tube wall. The boxes framed the location of the specimens for detailed analysis.

The macroscopic view of the failed tube is shown in Figure 2. The entire tube can be divided into four regions based on morphological features. In the non-crack region, no visible cracks or corrosion products are observed. In the sedimentary region, white deposits cover the tube. In the heavily cracked region, the three largest main cracks disperse uniformly with visible branches in the transverse direction. In the cracked region, nine cracks propagate along the circumferential direction. The four regions correspond to the four stages in corrosion failure. At the initial stage, the tube is relatively intact. As time elapses, the tube begins to be corroded and some corrosion products deposit on the surface. Then SCC initiates under deposits and propagates into heavy cracks. The cracks further develop and the tube ruptures. 

For further studying the characteristics of failure, specimens numbered from 1~5 were cut on different locations of the tube marked as ①~⑤, respectively. Specimens 1 were used for chemical composition tests, metallographic microstructure observation and electrochemical tests. Specimens 2 were used for XRD tests to analyze the composition of white deposits. Specimens 3 containing corrosion products were used for corrosion morphology observation. Specimens 4 were cut off to observe fracture morphology. Specimens with cracked tips numbered 5 were used for observing secondary cracks.

### 3.2. Material Inspection

The chemical composition of the 316 SS tube is shown in Table 1. According to the Chinese National Standard GB/T 20878-2007, the chemical composition is similar to that of S31608. However, the content of Ni and Mo is substandard compared with standard S31608, which may increase the tendency of sensitization and lower localized corrosion resistance, leading to corrosion and SCC [1,2,34].

**Figure 3:**
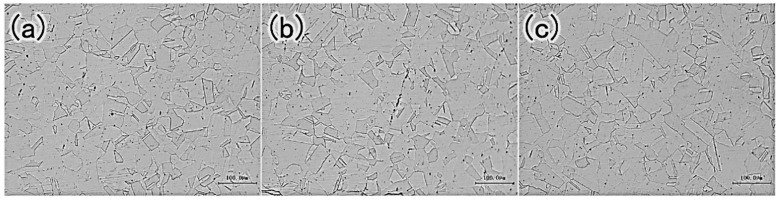
The metallography of tube at different zones (**a**) external tube, (**b**) middle tube and (**c**) internal tube.

The microstructure of the failed tube shows uniform austenite with annealing twins (Figure 3); the black-banded microstructure is δ ferrite attributed to friction during sample preparation process. No distinct carbides were observed along grain boundaries.

### 3.3. DL-EPR Test Results

**Figure 4:**
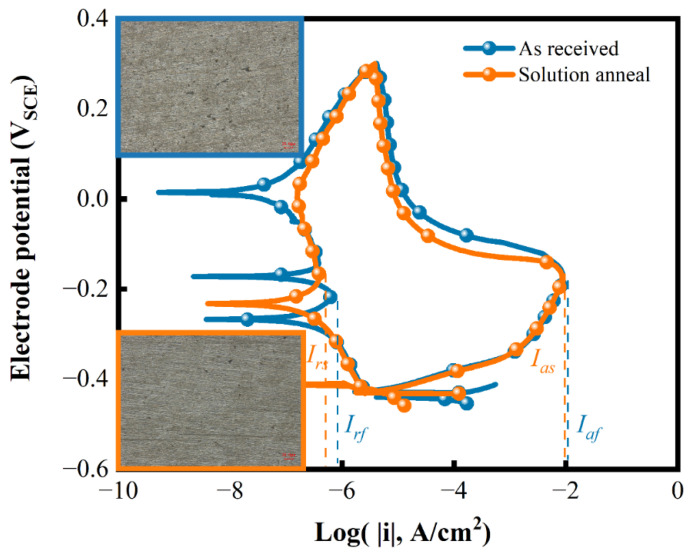
The DL-EPR test result of the as-received failed tube and the solution-annealed specimen.

The DL-EPR test was adopted to assess DoS quantitively. The result is shown in Figure 4. The forward scan activation curve of the as-received specimen almost coincides with that of the solution-annealed sample. Therefore, the critical potential and activation current to form passive film for both states is almost equal. Moreover, the reactivation peak in the reverse scan curve of both kinds of specimens is very small. Though two reactivation peaks appear during the reverse scan in the as-received specimen of the failed tube, the DoS of is no more than 1%, indicating no signs of sensitization to form Cr-depleted zones [35,36]. Therefore, the premature failure of the tube is not attributed to sensitization. The surface morphology after the DL-EPR test in the inset of Figure 4 is also consistent with this result.

### 3.4. Corrosion Analysis

**Figure 5:**
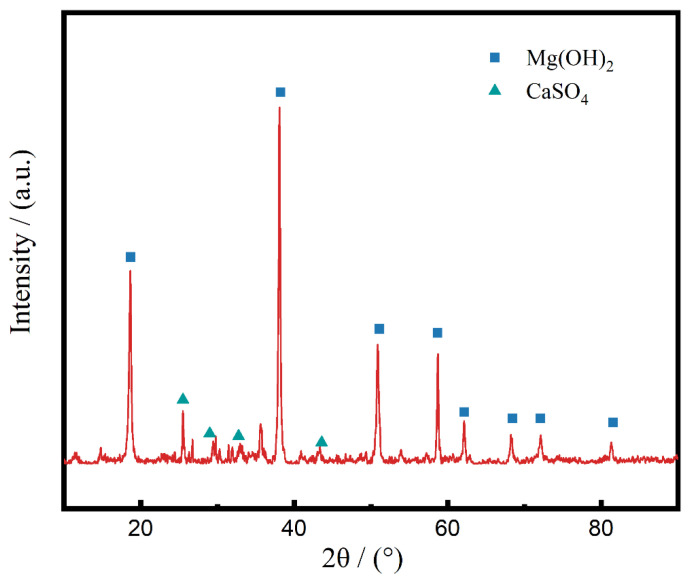
The XRD result of the white deposits.

The XRD result (Figure 5) shows the main composition of the white deposits consists of Mg(OH)_2_ and CaSO_4_.

**Figure 6:**
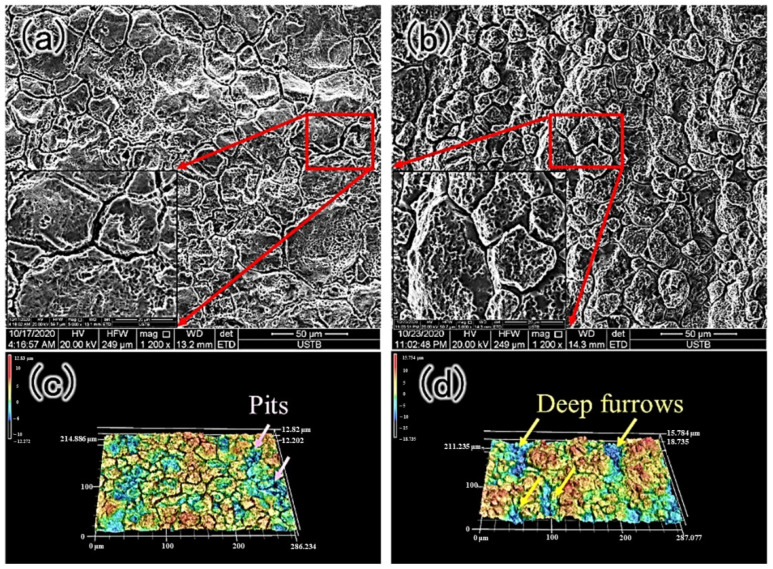
Corrosion morphology and the three-dimensional results. (**a**,**c**) correspond to the corrosion morphology of the external tube. (**b**,**d**) show the corrosion morphology of the internal tube.

After removing the corrosion products and deposits, corrosion morphology and three-dimensional results are shown in Figure 6. In the external surface, obvious intergranular corrosion morphology is apparent (Figure 6a). Three-dimensional corrosion morphology reveals the deepest localized corrosion is located at the intersection of grain boundaries (Figure 6c). The internal surface showed intergranular corrosion as well (Figure 6b), which manifest as deep furrows in the 3D profile (Figure 6d).

### 3.5. Fracture Morphology and Secondary Cracks

**Figure 7:**
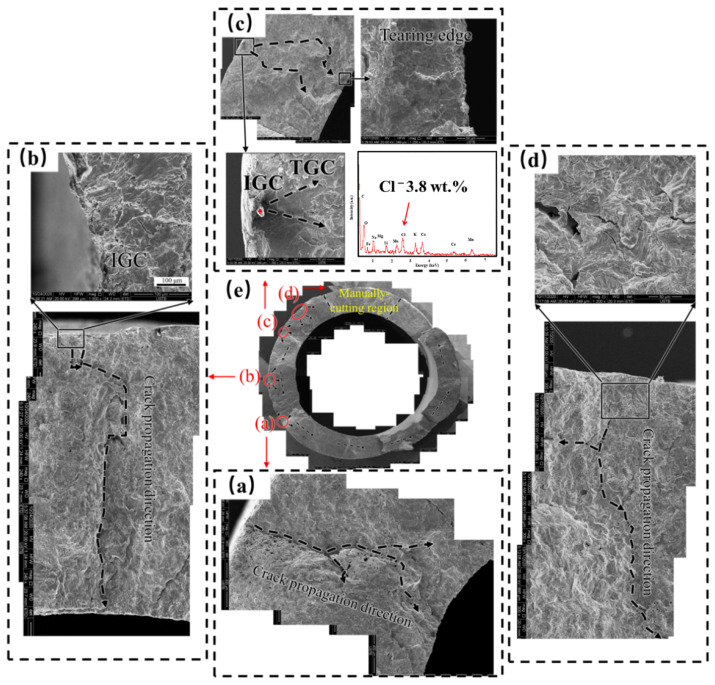
Fracture morphology in the main fracture. Four crack origins are shown in enlarged views in (**a**–**d**), and the fracture surface is shown in (**e**).

After removing the corrosion products, the fracture surface of sample 2 was shown in Figure 7. It should be noted that the black line circled region in Figure 7c is caused by a manual cut, which makes no difference to the failure cause and mechanism, so the morphology inside this region is not analyzed below. Through identifying the direction of radiation zones in Figure 7e, four crack initiation sites were identified; named zone (a), (b), (c) and (d) in Figure 7. In Figure 7a, the crack propagates in a herringbone pattern. In Figure 7b, the crack initiates from the external tube. Near the initiation site, secondary cracks along austenite grain boundaries penetrated through several grains. In the radial region, the crack propagated in the transgranular cracking (TGC) mode. In Figure 7c, the crack initiates from the external tube judging from the convergence direction of radial ridges. There is a cluster of white sediment at this site. After being examined by EDS, it shows 3.8 wt.% chlorides concentration. Within three grains away from the external tube, the cracking mode is intergranular cracking (IGC). However, outside this region, the cracking shows TGC morphology. Branching cracks are apparent, which are typical of SCC cracks. In the instantaneous fracture zone near the internal tube, tearing ridges of about 100 μm exist. Therefore, the multiple origination and propagation of microcracks induce fractures in Region 4 as Figure 2 shows.

In summary, there are three typical characteristics of the fractured surface. First, the cracks are branched transversely. Second, all the cracks initiate from the external tube, and some chlorides can be tested. Third, the crack initiation sites show IGC morphology or TGC morphology. Therefore, it can be deduced that fracturing is attributed to SCC. SCC for austenitic stainless steel is mainly related to the sensitive materials and corrosive environments. Sensitive materials include factors such as sensitization and a lack of corrosion-resistant elements [37,38,39]. The corrosive environments for austenite stainless steel contain aggressive ions, such as fluoride ions and chloride ions [4,40]. To reveal the cause of SCC, the above factors will be analyzed in the discussion part.

**Figure 8:**
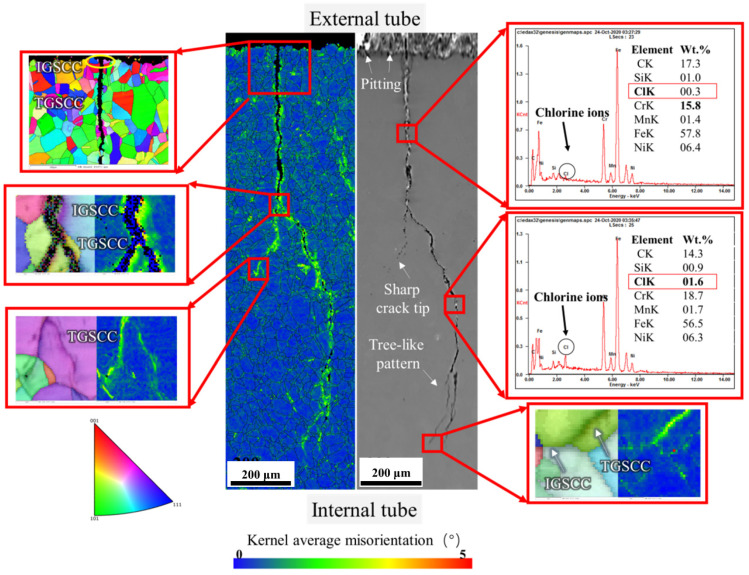
The EBSD result of an entire secondary crack.

To reveal the SCC propagation mode and the correlation of the crystallographic features, EBSD was conducted on an entire secondary crack, and the result is shown in Figure 8. The crack presents dendritic morphology. This morphology is consistent with the fracture surface in Figure 7. The crack initiates from the external tube in the IGC mode. Then the crack propagates mainly in the TGC mode. At the bifurcation position, it forked in the mixed mode of IGC and TGC. The chemical composition of corrosion products embedded in the crack was detected. Results show that chlorides can be tested (0.26 wt.% and 1.64 wt.%). Moreover, the crack tip is sharp, indicating that the tube is susceptible to SCC in the service environment [41,42]. The crack propagates in the mixed mode of IGC and TGC. The kernel average misorientation map (KAM) shows obviously high KAM values along the crack wall, especially at the crack tips. KAM represents the degree of local lattice distortion [43]. The high local lattice distortion along the crack may come from the swelling force of corrosion products and hydrogen-induced deformation attributed to hydrogen evolution reaction at the occluded crack environment [44]. 

**Figure 9:**
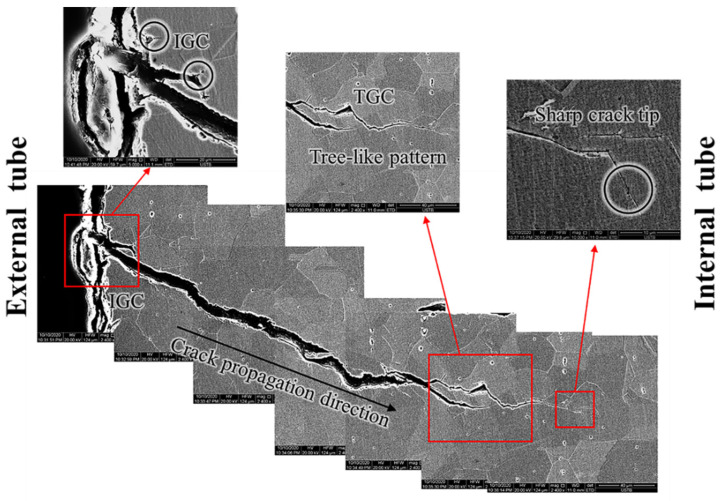
The cross-sectional morphology of a crack.

The cross-section morphology of another secondary crack is shown in Figure 9. The crack propagates from the external tube to the internal tube. In the area adjacent to the external tube, microcracks are intergranular, and the cracking mode changes to TGC when propagating to some extent. Moreover, the enlarged views show highly branching morphology and a sharp crack tip. This is consistent with the secondary crack in Figure 7 and Figure 8.

## 4. Discussion

### 4.1. Failure Causes

**Figure 10:**
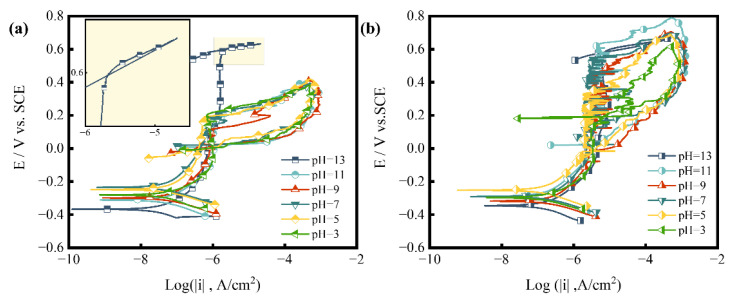
The cyclic polarization results tested in 0.1 mol/L NaCl at ambient temperature (**a**) the failed pipe, (**b**) standard S31608 SS.

**Figure 11:**
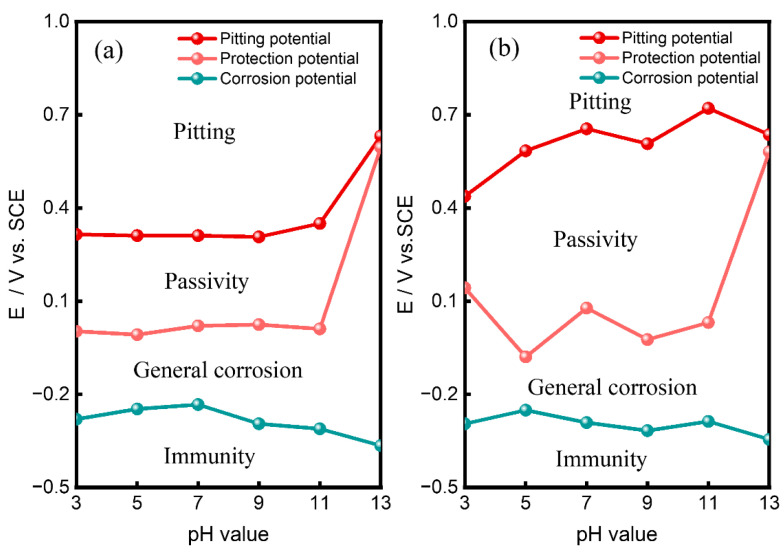
The potential-pH maps of (**a**) the failed tube and (**b**) standard S31608 SS in 0.1 mol/L NaCl.

The highly branching cracks are typical characteristics of SCC. Therefore, we can confirm that SCC is the main cause of failure. SCC is mainly dependent on three factors: material, environment and stress. For the material factor, the microstructure and DL-EPR results show no signs of sensitization, while chemical composition analysis reveals a lack of nickel and molybdenum compared with standard S31608 (Table 1). Nickel facilitates the formation of stable passive film and improves corrosion resistance [45,46]. Molybdenum has been reported to enrich the inner layer of passive film and benefit protection of passive film against corrosive ions [47,48]. Both elements can improve the pitting resistance of stainless steel. To quantitatively characterize the influence of nickel and molybdenum on the pitting resistance, the cyclic polarization tests of the tube material and standard S31608 in 0.1 mol/L NaCl solution at various pH values were examined as a comparison (Figure 10). The experimental potential-pH map according to cyclic polarization results is shown in Figure 11. Compared with standard S31608, the pitting potential of the tube is lower than over 100 mV at pH of 3–10. The reduction of the pitting potential indicates that the passive film of the tube material is easily destroyed and difficult to restore.

Corrosive environments also play an important role in SCC failure. The existence of chlorides can be detected in the fracture surface (Figure 7) and in the SCC secondary crack (Figure 8), in spite of the removal of corrosion products. Therefore, SCC can be accelerated due to chlorides. Corrosion morphology observation shows intergranular corrosion morphology (Figure 6), although the DoS of the failed tube is low (Figure 9). This phenomenon seems inconsistent with the traditional Cr-depletion mechanism induced by Cr_23_C_6_ precipitation on grain boundaries [49,50]. As Hu et al. [51] suggested, the co-segregation of Cr and C could also induce the intergranular corrosion of stainless steel without Cr-carbide precipitation. This theory could interpret the intergranular corrosion behavior in this study. For the stress, residual stress resulting from thermal stress between the internal tube and the external tube may account for the corrosion initiation stage. The swelling force of corrosion products and hydrogen induced deformation may contribute to the propagation stage.

**Figure 12:**
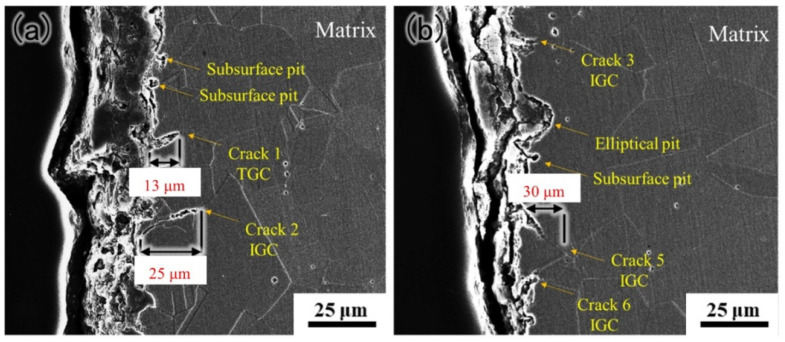
The cross-section morphologies of external tube at two different locations (**a**) and (**b**).

The cross-section morphology of the external tube shown in Figure 12 could reveal the early stage of the failure. Microcracks propagate as TGC and IGC. Meanwhile, elliptical pits and subsurface pits are observed inside grains. The penetration depth of microcracks is higher than pits. Based on the above results, the failure process may reappear. 

### 4.2. SCC Mechanism Analysis

Based on the above results and discussions, the SCC mechanism was proposed. The chloride ions in the service environment penetrate the passive film and promote the film breakdown. Intergranular corrosion occurs due to the co-segregation of grain boundaries. With residual stress, SCC microcracks are initiated. The occlusion effect at the crack tip increases the localized concentration of chloride ions. The lack of nickel and molybdenum destabilizes the passive film and reduces the resistance to crack propagation. Therefore, microcracks propagate. Because austenitic stainless steel has multiple dislocation slip systems, slips occurred under low stress intensity factor [52]. Therefore, the crack mainly extends in the transgranular mode. When the stress intensity factor exceeds the stress corrosion intensity factor, microcracks propagate spontaneously, coalesce into macrocracks and induce final rupture (Figure 2). On the whole, the stress corrosion cracking mechanism is a localized anodic dissolution (AD) mechanism. 

## 5. Conclusions and Further Suggestions

In this study, the premature failure of a 316 stainless steel was thoroughly investigated. The cracking type in this failure is chloride-induced stress corrosion cracking. Crack analysis and fracture analysis show that SCC originates from the external tube, propagates with branches and coalesces to cause the rupture. Chemical composition analysis shows a lack of nickel and molybdenum, which is detrimental to passivity. Corrosion analysis reveals chlorides in the service environment. From the above results, the SCC mechanism is confirmed as localized AD. On the microscopic scale, the cracking process includes the following processes: passive film rupture, chloride accumulation, SCC initiation and propagation. Based on the conclusions, further suggestions are made for preventing such SCC failures:

- Before service, verify whether the composition and microstructure of the steel parts meet the standard.
- Replace the 316 SS with more SCC resistant steels, such as 2205 duplex stainless steel.
- Reduce the concentration of chloride ions in industrial condensed water.
- Relieve the residual stress of the tube through solution treatment.

## Figures and Tables

**Table 1:** The chemical composition (wt.%) of the failed tube and standard S31608.

Element	C	Si	Mn	P	S	Ni	Cr	Mo
Failed pipe	0.042	0.47	1.42	0.033	0.0054	8.02	17.53	0.18
S31608	<0.08	<1.00	<2.00	<0.045	<0.030	10.00–14.00	16.00–18.00	2.00–3.00

Note: The chemical composition of S31608 is according to GB/T 20878-2007.

## Data Availability

Not applicable.
